# Electrophysiological insights into resilient individuals: Examining calcium permeable AMPA receptor responses and neuropathological correlations in Alzheimer’s Disease

**DOI:** 10.1002/alz.093208

**Published:** 2025-01-03

**Authors:** Berenice A Gutierrez, Naomi Moreno, C Dirk Keene, Agenor Limon

**Affiliations:** ^1^ The University of Texas Medical Branch, Galveston, TX USA; ^2^ University of Washington, School of Medicine, Seattle, WA USA; ^3^ University of Texas Medical Branch, Galveston, TX USA

## Abstract

**Background:**

Alzheimer’s disease (AD) is a common form of dementia characterized by the accumulation of amyloid beta (Aβ) and phosphorylated tau proteins in the brain. While clinical observations are typically used for AD diagnosis, postmortem studies have revealed individuals without dementia symptoms but with high AD pathology, known as resilient individuals. Calcium permeable AMPA receptors (CP‐AMPARs) have been implicated in the calcium dyshomeostasis of AD, but it is unclear whether they are found or behave differently at the electrophysiological level in resilient and control individuals compared to AD patients.

**Method:**

We examined the differences in responses of synaptic CP‐AMPARs from a cohort of postmortem tissue from 30 individuals, including controls (3M, 4F; non‐demented, no neuropathology), age‐matched AD patients (2M, 5F; individuals with AD of the same age as the control group), resilient individuals (2M, 6F; non‐demented with AD neuropathology), and age‐matched AD patients to resilient individuals (2M, 5F; individuals with AD of the same age as the resilient group). We isolated and microtransplanted synaptic membranes from the parietal cortex of each individual into *Xenopus* laevis oocytes for recordings of AMPARs through two electrode voltage clamp. We induced agonist responses of AMPARs with the use of kainate and glutamate drugs. To differentiate CP‐AMPARs from calcium‐impermeable AMPARs, we used IEM‐1460 drug, which selectively targets GluR2 subunit‐lacking receptors (CP‐AMPARs) over GluA2‐containing receptors (calcium‐impermeable AMPARs). Additionally, we performed immunoblots from the synaptosomal aliquots of each individual to assess the presence of Aβ/Tau oligomers and determine whether there is a correlation between the amount of toxic oligomers and the strength of the CP‐AMPARs responses

**Result:**

Our results suggest that resilient individuals tend to have less CP‐AMPARs synaptic responses compared to individuals with AD and even controls, despite the presence of Aβ/Tau oligomers.

**Conclusion:**

Understanding the differences in synaptic receptors among different groups of individuals provides a foundation for future research into the mechanisms that lead to synaptic alterations in AD.

